# Second International Conference on Publication Ethics organized by Shiraz University of Medical Sciences (Shiraz - Iran December 4-5, 2014)

**DOI:** 10.12669/pjms.312.7313

**Published:** 2015

**Authors:** Shaukat Ali Jawaid

Shiraz (Iran): Shiraz University of Medical Sciences (SUMS) in partnership with Committee on Publication Ethics (COPE) UK organized a two day Second International Congress on Publication Ethics at Shiraz from December 4-5^th^ 2014. It was held in collaboration with Iranian Medical Journals Commission, Iranian Society of Medical Editors and National Committee for Ethics in Medical Research

**Dr. Behrooz Astaneh** Chairperson of the organizing committee in his welcome address said that the number of scientific journals including medical journals has increased during the last five years and publication ethics has emerged as an important issue for the Medical Editors. We need to understand how scientific misconduct can be tackled. Iranian Ministry of Health is laying emphasis on quality of publications and research. Such conferences help educate the editors how to manage when they are faced with issues of scientific misconduct and publication ethics.

**Prof. Mohammad Hadi Imanieh**, Chancellor of Shiraz University of Medical Sciences in his speech said that SUMS has over ten thousand students and it was one of the leading universities in Iran. Realizing the importance of ethics, we have journals committee office with established ethics. We monitor the university journals with respect to publication ethics and standards. We provide training to the Editors and other staff. Such training programmes are essential for all editors in this region. These training programmes should be goal oriented and COPE role was extremely important in this regard. Since the number of journals in Iran is increasing our priority is their quality. Education of faculty as well as editors will help improve publication ethics, he added.

**Dr. Syed Bashir Hashemi** Vice Chancellor of Research Affairs at Shiraz University of Medical Sciences hoped that the participants will learn from each other during the discussions.

**Dr. Reza Malekzadeh**, Undersecretary for Research and Technology, Ministry of Health and Medical Education, Government of Iran in his speech pointed out that the number of retraction of manuscripts on PubMed as well as in ISI Thompson/Reuter Web of Sciences is increasing every year and the main reason for retraction is scientific misconduct. In many instances even scientists have plagiarized for their PhD Thesis. Plagiarism has become a very serious issue, we need to think about it and take action.

**Scientific misconduct is the main reason for increased retractions in PubMed & ISI-Dr. Reza Malekzadeh**

Publication Ethics, Dr. Malekzadeh opined was an important topic and participants will benefit from the discussion. We give due importance to publication ethics in the Ministry of Health and this issue is also highlighted by the media regularly. During the last ten years, the number of graduates as well as postgraduates in Iran, he said, has increased manifold which has also saw an increased number of publications. The number of publications is increasing from Saudi Arabia, Iran, Turkey as well as Egypt over the Years. Iran has made a major contribution to ISI Web of Sciences from the Muslim countries and during 2013, Iran ranked 17^th^ in the world as regards its number of documents for all science in Scopus database. Again during 2013, Iran’s ranking was 24^th^ as regards the number of Citations while 19.76% of the publications were with international collaboration. If we compare the Ranking of Iran with other countries in Middle East and Africa in Scopus database during 2013, Iran ranked No.1.

Continuing Dr. Malekzadeh said that the number of biomedical journals published from Iran also increased from 90 in 2004 to 345 in 2014. All these journals follow double blind peer review system, 95% of them have active functional websites, 186 journals have English full text and about twenty thousand papers are published every year from Iran. Giving further details Dr. Malekzadeh said that almost 32% of these documents are based on MS Thesis by the postgraduates and 28% of highly cited papers from Iran are also from medical sciences. About 40% of authors have no citation or just one citation which means that these manuscripts, studies had no impact. The number of Journals covered by PubMed from Iran is 72; those covered by EMBASE are 78 and indexed by Scopus are 91. Almost 24% of all journals published from Iran are in medical sciences. Some of the major challenges which these journals face are of infrastructure, lack of professionals, delay in peer review, and low citations besides publication ethics.

Referring to the help and assistance provided by the Iranian Ministry of Health Commission on Medical Journals, Dr. Malekzadeh mentioned checking title before publication, accreditation and Re-accreditation, provision of technical and financial support, monitoring and ranking, quality improvement, indexing, training of staff, preparing of websites besides having a central journal database.

Iranian Registry of Clinical Trials, he said, was established on December 4^th^ 2008. It is the responsibility of the research community to adhere to code of ethics and do honest reporting of the results of these trials. Usually there is a tendency to distort evidence, facts and go for positive findings. Financial interests also create a reporting bias. It was in September 2004 that the ICMJE took an initiative to publish only publicly registered trials. WHO also endorsed this initiative? WHO policy says that all interventional trials must be registered and everything must be publicly disclosed to ensure transparency. The number of clinical trials registered in Iranian Registry has increased over the years and till October 2014 it accounted for 1525 registrations and the number of trials registered during 2014 were 435. Dr. Malekzadeh was of the view that registration of trials is an ethical necessity. He also disclosed that in the MOH they intend to do more advance auditing of these trials besides monitoring the quality of these clinical trials.

Speaking about research misconduct Dr. Malekzadeh said that there is much focus on publication fraud. In most cases research misconduct starts before the paper is written and it is detected only when it is published which emphasizes the importance of publication misconduct. Such misconduct, he further stated, usually involves some level of intent but it is very difficult to prove. There can be some “Honest Errors” or mistakes but there was no justification at all for such a “sloppy science” which can be damaging. Referring to the reasons for misconduct and unethical behaviour, he mentioned lack of knowledge about research and publication ethics, increasing pressure on researchers to publish besides financial inducements offered to the authors which encourage them to compromise their integrity besides promotion policy for clinicians, researchers in the university. Almost 29% of retractions in Medline, he stated, were due to honest error, in 11% of cases it was not possible to replicate the findings, misconduct i.e. plagiarism accounted for 28% while redundant publications were 17% and 5% due to other reasons which could not be specified. Korea, Pakistan and China offer cash awards to researchers which was yet another reason why the authors indulge in scientific misconduct to increase their publications. His prescription for preventing such misconduct was educating the journals and universities. Institutions, he opined, should have clear guidelines for responsible conduct in research not only for students but all scientists working in their institution. Having one or two courses in the medical schools on ethics, were not enough.

**COPE promotes integrity in research publications, offers guidelines for peer reviewers and urges editors to behave ethically - Dr. Charlotte**

Dr. Malekzadeh suggested that the senior investigators and mentors should talk to their trainees about the importance of good scientific practice besides proving as good role models. We must ensure zero tolerance environment and strict actions should be taken against those who violate these guidelines. Suspected cases of misconduct should be reported and the institutions must have a mechanism in place for fair investigations. Findings of these investigations should be made public and notified to all the stake holders.

**Dr. Payam Kabiri**, Head of Electronic Resources Provision at SUMS talked about Publication Ethics Research: Trends and Tools. He defined research misconduct as fabrication, falsification, or plagiarism in proposing, performing, or reviewing research or in reporting research. Talking about research misconduct he mentioned plagiarism, failure to obtain ethical approval, failure to admit missing data, plagiarism, willful distortion of data, fabrication of data or cases, eliminating data on side effects, Gifts and Ghost authorship, redundant publication besides failure to do adequate research. He was of the view that we need to study different aspects of research misconduct through scientific research. Not only that academic research should investigate the different reasons for and the trend of research misconduct in the present academic world.

Continuing Dr. Payam Kabiri said that effective research in publication ethics will lead to recognition of more issues and it will also help us in preventing, resolving and managing such cases. He then gave details of a study they had conducted to find out research misconduct for which a search strategy was designed and developed to find out related papers in Scopus. This is the largest citation database which covers over twenty thousand journals. The search strategy included title and abstract with key words at plagiarism, research misconduct, scientific misconduct, fraudulent data, ethics in publishing or publication ethics besides data falsification and ghost writing. The search done in December 2014 retrieved 8,724 papers. It showed that the number of published papers in the field of research misconduct has increased from 169 in 1998 to 698 in 2013. Medicine had the highest number of ghost publications which accounted for 3,754 followed by social sciences which accounted for 1588. Surprisingly most of these Ghost papers were published in Nature followed by Science the journals with highest Impact Factor. Many countries including USA, UK Germany, Australia, Canada and China have published papers on research misconduct. Iran published forty five related papers and six of them were published in Archives of Iranian Medicine followed by five published in Acta Medica Iranica. Tehran University of Medical Sciences, Dr. Payam Kabiri said, contributed sixteen papers on research misconduct. Pakistan Journal of Medial Sciences also published two papers on research misconduct during the Year 2013.

Giving details of five top cited papers Dr. Payam Kabiri said that one of the highly cited paper published in 2009 in International Journal of Cardiology entitled Ethical authorship and publishing, had a total 1616 citations of which self citations accounted for 1555 which means that total citations excluding self citations were only 61. The second highly cited paper entitled Ethics in authorship and publishing of scientific articles was also published in International journal of Cardiology in 2010. It had total citations of 669 of which self citations accounted for 632 which means that citations excluding self citation were just 37. Similarly the third and fourth highly cited papers also had majority of self citations while the fifth highly cited paper was published in Journal of Physiology in 2006. It had total citations of 395 which included just 13 self citations while the real citations excluding self citations were 382. Dr. Payam Kabiri concluded his presentation by giving details of Elsevier Publishing Ethics Resource Kit (PERK) and Springer Publishing Ethics for Journals which provide lot of useful information on the subject.

**Dr. Ehsan Shamsi** Secretary of National Committee for Ethics in Biomedical Research from Ministry of Health and Medical Education made a presentation on “Moral Psychology Theories: A conceptual Framework for Controlling Medical Research Misconduct”. He referred to ethical and moral knowledge, moral sensitivity, moral judgment, moral motivation, moral action and moral character. The first step in Ethical and moral knowledge, Dr. Ehsan opined, was introducing clear publication ethics norms followed by communicating publication ethics standards and checking the target groups understanding of the subject. He highlighted the importance of creating a sensitizing environment and the need for specification publications on Ethics standards to cover such cases. Academic moral character and moral action avoiding any type of scientific/publication misconduct, he stated, was extremely important.

Giving details of the national plan for implementing WHO’s Standards for Research Ethics Committee he mentioned about the establishment of Research Ethics Committees in all institutions, giving more independence to these RECs. These RECs will be empowered to supervise all research process including publications. Their transparent functioning will be ensured and all these RECs will be well connected nationally. It is also planned to have more qualified members for these RECs who are more responsible and ethically sensitive. We in the MOH also plan random checking of 10% of papers published by each medical university annually. We will give credit to those universities who detect and report scientific misconduct in published papers. In addition implementation of a disciplinary system and national database for those who commit scientific misconduct in biomedical research will also be established. The primary investigators and their supervisors will be held responsible for ensuring observing ethical standards in the conduct of research. Postgraduate students will be required to regularly report progress of PhD and Master projects. The university ethics committee which detects scientific misconduct will be required to report it and approval from Research Ethics Committees will be mandatory for all research projects including the projects undertaken by the students. Approval of research proposals by National Committee and having a National Portal for Research Ethics Observatory programme are also on the cards, he remarked.

Earlier in her introductory remarks **Ms. Sarah Masoumi** said that Shiraz is a center for Medical Journalism and related issues. We have worked hard to organize this congress. Sitting together, she said, is progress while working together is a Success.

**Do not totally rely on software’s for detecting plagiarism because human judgment is always needed-Behrooz Astaneh**

The first scientific session was chaired by Mr. Shaukat Ali Jawaid from Pakistan along with Dr. Payam Kabiri from Iran and Dr. Aminul Haque from Bangladesh. **Dr. Behrooz Astaneh** was the first speaker who talked about various types of ethics misconduct and mentioned authorship disputes, conflict of interest, double submissions and redundant publications besides plagiarism, data fabrication and data falsification. There are two reasons for misconduct; some people do it intentionally while others do it because of lack of knowledge. Many researchers, Dr. Behrooz Astaneh opined, do not know what is considered as misconduct. Not only that even many members of the Editorial Boards do not know the exact definition of various types of misconduct while many editors too have no knowledge about it. In order to educate the authors and faculty members we have so far organized over four hundred workshops in different parts of the country, he added.

Continuing Dr. Behrooz Astaneh said that many editors do not know how to manage scientific misconduct. However, training can do nothing for those who deliberately commit misconduct. Authorship, he said, should be defined in byline and the ICMJE has four criteria for authorship which must be met by the authors. Defining the ghost author, he said that such person has the authorship criteria but is not included in the authors list. They could be students or junior researchers and it is not ethical. On the other hand guest author does not meet the authorship criteria but their names are added for prestige to increase the chances of publication. This too is unethical. These are mostly influential people, Head of the Department, Head of the institutions. Juniors put the name of their seniors which is also gift authorship.

He defined Conflict of interest as something when financial or personal relationship which inappropriately influences the actions of authors, editors or reviewers. This lack of disclosure can be unethical. Prior publication means publishing a full content of an article which has been published previously. However, abstracts, posters or oral presentation, Dr. Behrooz Astaneh clarified, does not come under prior publication. He further opined that internal meetings or report to investigators and regulators do not affect the later publication of such studies. In duplicate publications, the same data is published with identical text after making minor changes in authors order or title and abstract while redundant publication is anything which overlaps substantially with another publication. In this the same data is published with some changes in the text, different analysis is added or even some additional data is also added.

Speaking about reviewer’s misconduct he said that it refers to using confidential information of manuscripts referred for review to his/her own benefit by the reviewers. It also includes stealing the idea or data from the manuscript, rejecting a good quality manuscript or delaying publication of a manuscript from rival academicians because of professional jealousy or even not declaring potential conflict of interest.

## Plagiarism

It relates to using others intellectual properties without acknowledgement or making reference. This, Dr. Behrooz Astaneh said, was a spectrum to consider extent, originality, position, referencing, intention. His advice to the editor colleagues was not to totally rely on software’s for detecting plagiarism because human judgment is always needed.

## Data Fabrication

This was defined as making up data or results and recording or reporting them. This, he opined, is less prevalent than various other types of research misconduct but even then it is unacceptable to any extent. In such cases, there are no honest errors, he stated.

## Data Falsification

It means manipulating the already existed real data by omitting the undesirable one. Image manipulation is also included in this category. It also covers any attempt to alter or enhance the quality of an image in order to present the image factitiously better.

**Dr. Charlotte Haug** Vice President of COPE from Norway presented an overview and frequency of various misconducts. COPE, she stated was started in 1997 by a small group of Editors and at present it has over nine thousand members. Membership is open to all the editors from all subjects. COPE offers advice to editors, publishers but it does not investigate cases. It is a forum to discuss individual cases and all COPE members are supposed to follow the code of conduct for journal editors. COPE provides education, guidelines and advice to its members. Flow charts prepared by COPE cover many things and it is all available on its website. She then talked about publication ethics and said that cases which are discussed in COPE forum are actual cases. It is a great resource for learning. From 2009-2012, over six hundred cases were discussed and their details are on COPE website. She was of the view that over the years the issues have become more complicated. She also gave details of classification of cases discussed in COPE forum. COPE, Dr. Charlotte said promotes integrity in research publications, offers guidelines for peer reviewers and urges editors to behave ethically. Whatever the editors know is all confidential and they are not supposed to use this data for themselves. Editors have responsibility for peer reviewers. All cases discussed in the COPE Forum are entered into the database, no name is used and it is a good learning resource, she concluded.

**COPE offers advice to editors, publishers but it does not investigate cases - Dr. Charlotte Haug**

**Ms. Lida Mokhtari** talked about regional COPE membership. She pointed out that there has been an increase in the number of journals from Iran and at present 142 are members of COPE. It includes 99 medical journals. So far we have organized 425 workshops where COPE was represented. Majority of the English journals in the region, she said, do not know about COPE. Many journals in the region are facing ethical problems and it was a challenging time for the journals and publishes. Lack of knowledge about COPE, lower representation of COPE in various countries because of a few members was also highlighted. She urged all the journals to sign up, join COPE and follow the code of conduct for the journals.

**Dr. Fatema Jawad** Chief Editor of JPMA from Pakistan highlighted the problems faced by the editors to ascertain who the real authors are. People, she said, undertake research to discover new therapies, they write for promotions, to beautify their CV, influence others, and gain recognition. However, some of these authors also have a psychological problem. One that originates or creates research is known as author or is entitled to be an author. Continuing Dr. Fatema Jawad said that in the good old day’s single person used to conceive the idea and write a paper but now there are layers of authors. All those who conceive the idea, get it approved, those who actually perform research, those who analyze the data, those involved in literature search, those who finally prepare the manuscript and others who give their blessings and give final approval of the manuscript to be published are all included in authorship.

Every institution must have an ERC/ERB to supervise research; its members should have adequate knowledge, experience of research and publication ethics- Dr. Fatema Jawad

Continuing Dr.Fatema Jawad said that all authors have equal responsibility towards conducting research and its publication. It is essential that the policy of the respective institution on authorship should be known to all and it must be followed. Ethical principles should be followed by all like conflict of interest should be disclosed; study design and safety of the research subjects should also be ensured. All authors are supposed to meet all the four criteria for authorship as laid down by ICMJE. However, it is the primary author who is responsible for entire research and he/she should also be responsible for contribution of other authors. It is also important that individual contribution of each author is known and agreed upon among the authors but it must be convincing.

From an Editor’s point of view, the qualities of an author, Dr. Fatema Jawad said include good writing, accuracy, knowledge on context and citations, no hesitation to publish negative results, clear understanding of conflict of interest issues as well as acknowledgement, understanding the copy rights law and he/should also be fully conversant with ethical issues by COPE and ICMJE. Someone who supports research, arranges funding, provides technical services, involved in collection of data of patients or from laboratory material alone does not deserve to be included as authors. Many a times the authors offer gift authorship in appreciation or respect of an eminent, important personality which can increase the credibility besides improving the chances of publication. Authorship can also be earned through coercion; it is also demanded by colleagues who wish to have more publications. She then presented details of three cases wherein the student who conducted research was warned of dire consequences if the name of the supervisor was not added as No. 1 author. The other case involved two students’ projects which were published online with the supervisor as the first author. In the third case the reason given was that the study was too good to be done by students, hence the name of the supervisor who took the soft copy and changed the order of authors was listed as No. 1 author.

Continuing Dr.Fatema Jawad said that we have noticed that sometimes order of name of authors is suddenly changed; a name is deleted or added during revision without any notice. Faculty members need publications for promotion despite the fact that many of them do not have the basic knowledge of conducting research. They are too busy clinicians and have not time for research. Hence the easy method they adopt is to involve the students who are more knowledgeable, energetic and eager to learn and are also more competent in the use of computers. Hence it is important that each revised manuscript is checked carefully as regards authors and their numbering and correspondence is limited to the correspondence authors.

She suggested that each institution must have an Ethic Research Committee/Board to supervise research; members of the ERBs should have adequate knowledge, experience of research and publication ethics. Further more there should be uniform guidelines for conducting research and publications which should be implemented. The students and faculty members must know these ethical principles and follow them and punishment for any malpractice should also be clearly specified. She concluded her presentation by stating that research was essential for progress while publication of research was an obligation. Authors can resort to unethical measures to get their manuscripts published. As such it is essential that editors should have good knowledge of publication ethics and implement them. Above all editors and editorial staff has to be vigilant and do not Trust anyone blindly.

**Dr. M. Mallaei** from Iran shared the results of their study regarding views of faculty members and staff of research centers about the ICMJE authorship criteria. Data was collected through questionnaire from sixty six faculty members and staff of eight research centers besides Vice Chancellors for Research. Most of the participants had one to forty papers to their credit. Forty four had attended a workshop on medical journalism. Only eleven claimed that they were aware about the ICMJE authorship criteria but only seven could correctly state it. Thirteen felt that all the four criteria must be met to be eligible for authorship. Those who had attended the workshop or had knowledge of Codes of MOH about publication ethics were better informed. Thirty eight felt that they were not included as authors while they did deserve that while twenty said they did not deserve authorship but were still included. Fourteen were not aware that they have been included as authors while forty seven said their placement in authorship list was not proper.

During the discussion many ethical issues were highlighted. Students it was stated should be included as authors when they have done the experiment. It is essential that we help the students. Conducting research and preparing the manuscript these days is a team work and all those who have made some intellectual contribution must be included as authors. Ideally the issue of authorship and placement of authors should be decided before starting the study and submission of the manuscript. Change in authorship and their placement must be discouraged later on unless all the listed authors agree in writing to any such change.

Making the best use of information technology organizers of the conference arranged presentations through Video Conferencing from London and Oxford in the second session on December 4^th^ 2014. This session was chaired by Dr. Charlotte Haug, Dr. Fatema Jawad and Dr. Ehsan Shamsi. **Dr. Trish Groves** Head of Research at BMJ and Editor-in-Chief of BMJ Open made a presentation on Authorship Criteria: Why the fourth criterion was needed?

She pointed out that research when completed must be fully reported. ICMJE is a small working group and we meet every year. Authorship matters because it confers credit important for academics but one is also responsible for the published work. It means responsibility and accountability. Fourth criteria was added to the ICMJE guidelines on authorship to ensure that all authors must be responsible for integrity of the work. If any problem arises, all the authors are responsible to investigate it and solve those problems. All authors should identify co-authors who are responsible for specific parts of the work. Study group can have many authors but those should be listed who are involved in most of the work. It should clearly state the contributorship as to who did what. Others also contributed to the study but they did not qualify for authorship but they all must be acknowledged. In transparency declaration the lead author confirms that no important aspects have been omitted and everything has been explained.

During the discussion it was pointed out that while including the fourth criteria in ICMJE guidelines, the title of the guidelines was also changed. These are now recommendations and it is expected that every journal and every institution follows it and gets it implemented.

The next presentation through video conferencing was by **Prof. Douglas Altman** from Centre for Statistics and medicine from Oxford University. His topic was adhering to CONSORT statement in reporting RCTs. He was of the view that research has validity if methods have validity and research findings are published in usable form. Speaking about the RCTs as to what has happened he referred to the WMA as well as Helsinki declaration. It is the obligation to conduct research ethically and report honestly. It is important that all trials are registered and trial results are made publicly available within reasonable time of completion of the study.

Speaking about taxonomy of poor reporting he said it relates to non-publication or selective reporting or incomplete reporting. It is misleading reporting to make the results simply positive. These practices, he opined, are very common. Referring to the harm of poor reporting he said that it leads to over estimation of advantages and the given treatment. In one of the studies in 164 trials, 31% had bias in reporting while in another non-pharmacological intervention study 39% out of 137 had reporting bias. In many systematic reviews the readers complain that they cannot extract from the publication what does it mean? Poor reporting is a serious problem in systematic reviews. Speaking as to what can be done to improve this situation, Prof. Douglas said that there is a responsibility of every one involved to ensure that published research is an unbiased. All trials are registered and all results are reported. In reality many trials are never published. He further stated that authors should sign a declaration of transparency and conflict of interest. CONSORT has a 25-item check list stating what should be reported in a paper. It should also contain a flow diagram describing patient progress which should be included in trial report. Many journals, he said, has adopted this Consort statement. He concluded his presentation by stating that not all trials are registered and not all trials are published. Journal articles are seriously inadequate and improvement overtime is slow. UK research system requires that researchers will adhere to Helsinki declaration. Many journals have included this in their instructions to authors but authors are not reading and following these guidelines. He emphasized the need to strengthening the reporting culture. It is essential that Ethics Committees, scientists, organizations, editors all adhere to it and work together. He recommended that completeness, accuracy was need of the society at large. We must make authors to follow the reporting systems, look at the manuscript carefully, and support registration for publication besides training peer reviewers. Trial reporting should be completely transparent and all parties need to remedy this unacceptable situation, he remarked.

**All trials should be registered and all results reported but in reality many trials are never published - Prof. Douglas Altman**

**Dr. Ponneh Sarveravan** from Iran gave details about adhering to CONSORT statement in RCTs with pharmaceutical interventions. They included 492 pharmacological RCTs which met the inclusion criteria. Of these 280 were published in Persian language and 230 in English language and less then 50% adhered to CONSORT statement. Three hundred seventy six had identified RCTs in the title, 445 completely defined the pre-specified secondary outcome measures, 326 gave details regarding the methods used to generate or random allocation. 429 gave details regarding mechanism used to implement the random allocation, 480 mentioned who generated the random allocation, 489 listed as to who assigned participants to interventions. Registration number and name of the trial registry was mentioned by 297 and 339 RCTs respectively while 493 gave information as to where the full trial protocol can be accessed. Their conclusions were that RCTs published in Iranian medical journals do not adhere well to CONSORT statement. The editors, authors and reviewers all need to be trained to consider CONSORT statement in reporting clinical trials well.

**Mr. Shaukat Ali Jawaid** from Pakistan in his presentation pointed out that authors are the most dangerous pressure groups which the Editors of biomedical journals have to face. The situation is worse in developing Third world countries where the authors, academicians are under tremendous pressure to publish for academic advancements and promotions. He supported his statements by referring to the various e mails received from the authors. He advised his editor colleagues to be careful when they receive too much submission from the same e mail, do not entertain manuscripts from professional groups, business groups but deal with the authors directly. Always ensure that the submitted manuscripts are accompanied by Ethics Committee/IRB approval. In case of submissions from overseas if you suspect the signatures on letter of undertaking to be suspicious, ask the authors to resubmit the LOU with proper signatures. Check the ménace of gift authorship, in case of too many authors, ask the submitters to reduce the number of authors, have a good peer review system. He further suggested that one should maintain record of correspondence with authors and reviewers for at least two years, always screen manuscripts for plagiarism, create awareness about scientific misconduct, go for CME and Continuous Professional Development which is the key to success and learn from colleagues how to face certain difficult situations. Presentation by **Prof. Aminul Haque** from Bangladesh was based on the ICMJE criteria for authorship which is available on the ICMJE website.

**Authors are the most dangerous pressure groups which the Editors of biomedical journals have to face- Shaukat Ali Jawaid**

What constitutes authorship was discussed by **Dr. Zoe Mullan**, Editor of Lancet Global Health. She described in detail the common problems encountered and the current definition of authorship, advantages of authorship. She also disclosed that there are 877 cases related to authorship on the COPE website. COPE has also developed six flow charts related to authorship. ICMJE as well as American Physiological Society all give importance to concept, design of the study, significant contribution. Execution of study or interpretation is open to question but ICMJE guidelines are stricter in this regard. She further stated that it is not for the Editors to decide whether someone is or is not eligible to be an author. Every journal, she opined, should define authorship policy and all authors must sign authorship statement. Editors should ask for details regarding contributorship besides a declaration that the authors take the responsibility for integrity of the research work. She also talked about acknowledgment and said that those who meet some criteria but not all the four can be listed in acknowledgement. COPE recommends that individuals so named should also sign a declaration of agreement. The editors should also consider sending correspondence about a submitted paper to all named authors to reduce the possibility that some individuals may have been included without their consent. At times problems do arise and it is the responsibility of the editors to get it resolved. Do not feel that you must make the judgment yourself instead always refer to the authors instructions and COPE is there to help, she added.

**Dr. Mohammad Irfan** from Pakistan discussed regional view on authorship. He started his presentation with a case from Pakistan being discussed on the PAME List serve wherein the authorship was in dispute due to differences between the principal investigator and his supervisor. He pointed out that Supervisors are supposed to be mentors but in such a scenario, one can only feel pity for the young researchers/trainees. Even our religion says that one must have a mentor in life but if we consider the ground realities about mentorship, the situation is not promising. Referring to a survey in 2002 conducted among 4160 earlier career and 3,600 mid-career biomedical and social science researchers, he said that early career researches who received mentoring related to ethics and research decreased the odds of engaging in research misconduct but mentoring on professional survival increased these odds. About 2% of scientists admitted that they fabricated, falsified or modified the data, 77% admitted misconduct. Mentoring, Dr. Irfan said is important in the training and grooming of successive generation of scientists. A study among 92 Nobel laureates showed that more than half of them had worked under older Nobel laureates. What was transmitted to them was not just knowledge or skills but a style of thinking, he remarked. Talking about as to what constitutes inadequate mentoring he mentioned failure to review trainee raw data at regular intervals, failure to establish clear standards and failure to adequately support the trainee in career development. Talking about characteristics of successful mentoring relationship Dr. Irfan mentioned reciprocity, mutual respect and clear expectations.

**Mentoring is important in training and grooming of successive generation of scientists-Dr. Irfan**

Continuing Dr. Irfan said that mentoring fails because of poor communication, lack of commitment, personality differences, conflict of interest and lack of experience. Toxic mentors are destroyers or criticizers. The Dumpers are mentors who force novices into new roles and let them sink or swim while Blocker mentors are those who continually refuse requests, withhold information, take over projects or supervise too closely whereas the Avoiders are the mentors who are neither available nor accessible.

As regards regional mentorship, research culture, Dr. Irfan stated, has not yet developed, RCTs are not so frequently carried out, publications are essential for promotion and improving the CVs, training is inadequate an the time available is short. Sometimes the authors are forced to put the name of seniors in byline of their manuscripts though they have had no contribution at all. Sometimes reviewers deliberately ignore some shortcomings in a manuscript from his friend or former professor/boss. All these are the harsh facts which must be considered while proposing any guidelines for acceptable ethical behaviour. He was of the view that the situation can be improved by creating awareness about authorship guidelines, Ethical Approval certificates, acknowledgement of submission being sent to all authors and enhancing the role of the regulatory authorities like HEC in Pakistan through the respective institutions. Institutions can play a role if trainees have identified mentors, they are provided training on mentoring skills, provide resources and support for mentoring the faculty, have guidelines on best practices in mentoring besides recognizing and rewarding the excellence in mentoring, Dr. Irfan concluded.

**Authors who fulfill all the f our ICMJE criteria for authorship should get equal credit- Dr. Behrooz Astaneh**

Replying to a question during the discussion, **Dr. Behrooz Astaneh** said that there is a suggestion from the Iranian MOH that try not to publish studies in your own university journal and it was not being followed by some. Change in authorship should be decided in the beginning as the project starts. It should also be decided who will be the first, second and third author etc. One of the participants from Mashad University opined that PhD students will have problems if it is decided in the beginning. **Prof. Handjani** remarked that different countries have different credit system for first, second, third and other authors. Usually it is the first author who is expected to do most of the work. Some journals mention name of authors in alphabetical order and it is also specified in the instructions to authors. As such the Editors should not get involved in authorship listing. Dr. Behrooz Astaneh opined that all those authors who fulfill all the four ICMJE criteria for authorship should get equal credit. It was also emphasized that Journals should have an Appeal process for the authors whose manuscripts are rejected. **Shaukat Ali Jawaid** remarked that in their experience two authors appealed against the decisions of the Reviewers and when the authors challenged the decision with documentary evidence of latest research supporting their findings, their appeal was upheld and the reviewers also thanked them for the feedback and updating them. It shows that the authors who have done the study and prepared the manuscripts are most of the time much better informed than the reviewers. That is why to be a reviewer offers many advantages.

The first session on Day two of the conference was chaired by Dr. Mohammad Irfan from Pakistan and Dr. M. R. Panhehshahin from Iran. **Foirouzan Akrami** was the first speaker who discussed Iran’s challenges in protection of Copy Rights. It was stated that protection of intellectual property rights leads to job and growth of economy. He also emphasized the importance of capacity building, promotion of culture of early childhood education besides providing economic, cultural and legal infrastructure.

**Dr. Firoozeh Yaganeh** from Iran spoke about redundant publications among Iranian English Medical Journals. Duplication publication, it was stated means same hypothesis and same results while Salami slicing means splitting the data from the same study into more than one papers while redundant publications are waste of time and it also destroys the journal reputation. They looked at 480 manuscripts and found twenty five duplicate publication during 2006-2009 and 34 during 2010-2013 and the total duplication publications during these two periods were fifty nine which accounted for 6.1%. It may be a bit higher if one takes into account the articles published overseas by Iranian authors. It also showed that the authorities in Iran have been successful in checking duplicate publications through education and creating awareness about scientific misconduct.

**Dr. Parisa Khani** discussed frequency of reporting ethical protection in human subjects in manuscripts published in Iranian Journal of Medical Sciences. They reviewed 1460 human subject articles of which 52.9% were in Persian and the rest in English. 443 reported ethical approval, 686 mentioned about informed consent, 341 declared conflict of interest, 595 manuscripts mentioned about the financial support. It also showed that English Journal reporting was better and over the years it has made some improvement in all these areas.

**Prof. Farhad Handjani** talked about ethical advertisement policies of medical journals. He was of the view that the advertisements should carry clear information and it should also be evidence based. However, when the journals sell the reprints of the manuscripts published and make money, it brings in the conflict of interest. He further stated that it is important to carefully look at the quality and contents of the advertisements. Leading journals like BMJ and JAMA who are members of the ICMJE as well continue to get huge revenue from the advertisements. Advertisements must contain information regarding indications, contra indications, and dose but in practice, many of these advertisements make misleading claims. Prof. Farhad Handjani suggested that the medical journals should explore other sources of revenue than advertisements to minimize the conflict of interest. Participating in the discussion Mr. Shaukat Ali Jawaid from Pakistan remarked that in Pakistan when Tegaserod was launched, the company made exaggerated claims regarding its efficacy in IBS and all the gastroenterologists promoted this drug by name but eventually it had to be withdrawn after a year. He was of the view that it is not only the pharmaceutical companies but the members of the medical profession are also equally guilty in such unethical practices.

**Plagiarism cases are complicated, it is always better to be careful and think of consequences-Dr. Charlotte**

**Dr. Zoe Mullan** described Lancet experience on confronting ethical misconduct. In the first case she presented, was related to case series of twelve children with regressive development disorder and intestinal abnormalities. In eight children parents associated onset of symptoms with receipt of MMR vaccine. This paper was published in 1998 despite disenting remarks by two of the authors. In February 2004 the journal received allegations of lack of ethics approval, bias in the study design and conflict of interest. The journal asked all authors to respond to these allegations and also asked the institution concerned to conduct investigations. In March 2004, ten of twelve authors formally retracted interpreted association between MMR vaccine and the syndrome described. In January 2010 General Medical Council in its hearing found three authors were involved in ethical lapses, dishonesty and conflict of interest. Hence this manuscript was eventually retracted by the editors in February 2010.

What we learnt from this episode, she said, was that never publish such papers with small number of patients, descriptive findings which has the potential of misinterpretation. It was also decided to publish source of funding of such studies besides information about potential conflict of interest. She also described two more cases in detail and said that since then the journal now requires registration of randomized trials before the start of the study, submission of a pre-specified protocol for scrutiny with the paper and confirming the newly added fourth criteria for authorship by the ICMJE. We also learnt that one should contact as many bodies and institutions as possible for help and assistance in dealing such cases. If it is impossible to resolve, one should avoid withholding important data to ensure full transparency. She concluded her presentation by stating that one should take all alerts of possible misconduct and other ethical concerns seriously. Regular meetings to consider difficult cases, one should be open and transparent regarding editorial decisions and if still there are some doubts, the case should be referred to COPE for advice. Responding to a question as to why Lancet published the first case when two of the authors had dissenting notes, Dr. Zoe Mullan said, it is very rare. It was also pointed out that the Ethics Committee job is very difficult. In the past there was a trust between the editors and authors but unfortunately this trust is now being betrayed quite frequently.

In the second session which was chaired by Prof. Farhad Handjani, Behrooz Astaneh and Dr. Zoe Mullan, **Dr. Bibi Sedigheh** from Iran talked about inadvertent plagiarism and ethical misconduct. She was of the view that at times it is inadvertent due to over reliance on others. It is useful to know the reviewers. If the reviewers have been suggested by the authors, one should ask them about their published papers. She further stated that students should be taught how to avoid plagiarism. Many students do not realize the importance of time and leave the manuscript writing till the end and then they are under pressure to publish, hence the temptation to indulge in scientific misconduct. In another case one author submitted a manuscript to IJBMS and also suggested three reviewers. When favorable comments were received, it was detected that it was the author himself who had sent these comments but made them look as if they were from the recommended reviewers. The next presentation by **Ali Mohammad** and colleagues was on comparison of five plagiarism detection software’s. It was pointed out that iThenticate software was one of the best which is more accurate. It is superior as it also covers some other languages than English alone; it is also easy to use. He also talked about the salient features of other software’s like Plagiarism Checker X and Plagiarismdetection.org and pointed out that one should go into details of plagiarism instead of just looking at the similarity index score. **Dr. Ali Vahadani** from UAE spoke about research and publication ethics. Giving details about Iranian Red Crescent Medical Journal, he said that it is published by Iranian Hospital in Dubai and in 1998 its title was changed. Since 2011 it is a monthly publication and our manuscript processing time is one to three months. We use iThenticate software for detecting plagiarism. Best defense, he said, was to educate the authors, editors and reviewers. These guidelines need to be customized and improved for different regions.

**Training can do nothing for those who deliberately commit misconduct - Dr. Behrooz Astaneh**

**To conduct research ethically and report honestly is an obligation-Prof. Douglas Altman**

**Dr. Charlotte Haug** made a presentation on Corrections and Retraction. She referred to a Japanese researcher who claimed to have found an easy way to grow stem cells in his paper. This could not be replicated. When the institution was contacted, it was investigated and this paper was retracted in July this year. Eventually the author Yoshiki Sasai committed suicide. She then quoted Rennie D who had reported that science does not exist unless it is published. Publication is integral to research. Advance in science, she stated, is seldom made by single researcher but it is by various scientists who make the difference. She highlighted the importance of quality and integrity of scientific literature. The first retraction by authors was reported on June 24^th^ 1756 and since then the number of retractions has been going up. Since 1997, Science, Nature have made most of the retractions. Editors, Dr. Charlotte opined, are accountable and should take responsibility what they publish. When the editors have clear evidence that the findings are unreliable, the paper should be retracted. Such publications report unethical research. If findings have been published somewhere else without proper cross referencing, it also constitutes plagiarism.

Continuing Dr. Charlotte said that one should not retract a paper just to punish authors who misbehave if small part of the article reports flawed data. When change of authors is required but there is no reason to doubt the validity of the findings it should be accepted. Honest errors have to be corrected. Retraction notice, she said, should be linked to the retraction article and it should also be clearly defined as to why this manuscript was retracted. The best practice is let the authors retract themselves but if they do not, then the Editors should retract such manuscripts. The retractions do show up in the PubMed. She also referred to threats from the lawyers and in case of total silence from the authors; the editors have to find a way. Sometimes the publisher’s lawyers wish to retract the paper to avoid law suits.

**Ms. Sarah Masoumi** discussed ethical standards from the viewpoint of various indexing systems and emphasized the importance of the journal being indexed. As a result of international recognition, indexing in important databases, she said, will increase the visibility, will have increased submissions and reputation of the journal will also increase. ISI, Medscape, DOAJ, PubMed Central all have their own requirements for indexing of journals. It is related to quality, quantity, and some technical requirements.

Continuing Ms. Sarah Masoumi pointed out that ISI looks at timely publication, international editorial board, per review system, editorial contents, and citation analysis. Medline looks at scope, quality, international editorial board, production quality, types of contents, geographic coverage. PMC looks at scientific quality, technical evaluation, XML files but it has no ethical requirements and no check for ethical issues. DOAJ look at if the journals follow WAME and COPE guidelines. Scopus looks at contents, peer review, regularity of publication, reasonable contents, publication ethics and malpractice statement. Statement regarding following WAME, ICMJE and COPE guidelines should be mentioned. She also talked about PERK guidelines on publishing ethics. Index Copernicus looks at scientific quality, standard, printing quality, website scope, and editorial quality. Scopus was the only database which made publication ethics mandatory. WAME, ICMJE, COPE guidelines can be used and they are all very helpful. She further stated that Editors, Reviewers, Publishers and Society at large all are responsible for ethical behaviour. One should know how the cases suspected of scientific misconduct are to be investigated. There does exist some gap between the indexing systems and journals. Increased interaction between them and training of authors, editors as well as reviewers will bridge this gap, Sarah Masoumi remarked.

**Various countries have different credit system for authors hence Editors should not get involved in authorship listing-Prof. Farhad Handjani**

In the last scientific session **Dr. Majid Asadi Samani** speaking about internet scam and fraudulent publications said that one must pay attention to the website of the journals. Make sure that you have access to the past issues of the journal or contact the editor for verification. Evaluate the overall design of the website and if any one offers to publish your article fast, reject this offer.

**Dr. Leila Ghahremani** discussed difficulties of producing evidence based journalology. Editors, she said, are gate keepers of research and reviewers are supposed to increase the validity and quality of reports. To study the peer review system in Iran, they included 51 journals in the study. Editors were contacted every two weeks. One article was selected for open peer review and one for blind peer review. Nine editors felt that blind peer review is the best while five editors said that they have not yet taken a decision. Medical editors, it was stated, should pay more attention to improvement and publication. They want to improve the quality and they were also competing with citation of their journal. Dr.Behrooz Astaneh opined that Editors should compete to have evidence based for which we need co-operation to produce evidence.

**Editors are accountable and should take responsibility for what they publish-Dr. Charlotte**

**Dr. Trish Grove** from BMJ talked about ethical aspects of Open Access Publishing through video conferencing from London She discussed at length the advantages and disadvantages of open access and history of open access. The gold standard open access publishing is the documents published by the journal while green stands for self archiving of authors work including those accepted for publication. From 1992 to 2009, there were over five thousand open access journals. In predatory open access it is the authors who pay. Open access, she further stated, has given birth to many online publishers and many of them have no peer review system at all. She then referred to COPE principles of transparency in scholarly publishing. Peer review, process, she said, should be clearly explained. Editorial Board should be listed and editorial team contact information should be given. Author’s publication fee should also be clearly stated besides explaining the sources of revenue as well as the advertising policy. Publishing schedule, frequency of publication should also be clearly mentioned, she added.

During the discussion sanctions against Iran having an adverse impact were also discussed whereby ISI database was not giving access to Iran. COPE, it was stated, has a policy of more inclusive and allowing reasonable access to resources. Some of the participants felt that such issues like sanctions against Iran should be discussed by COPE members. However, COPE council members pointed out that it is an independent organization which does not interfere in administration. Journals should always look at the contents on the paper; there are some legal issues in which COPE was not at all involved.

**Prof. Lorraine Ferris** head of Ethics Committee of WAME made a presentation through video conferencing from Toronto and discussed Editors dealing with errors and allegations of research misconduct. This presentation highlighted the fact that editors have an important role in safeguarding integrity of scholarly publishing. They should not pass any judgment until all the facts have been collected. They should recognize the possibility of problems and be prepared, educated how to deal with such problems. They should stay involved in these issues till conclusion or decision is made. They should be fair, sensitive, respect both complainant and those against whom allegations are leveled. Provide both the parties an opportunity to respond, discuss both the responses before deciding about the next step. They must ensure to handle matters timely and take appropriate actions. Errors are mistakes, inaccuracies. Journal website, it was pointed out, should have definition of research misconduct. The editors should also ensure screening for plagiarism and image manipulation. Editors, it was further stated, have an important role in safeguarding integrity of scientific record.

**Omid Asemani** spoke about the right of intellectual possession or academic conventions to be the criteria of authorship while **Dr. Al-Taitoon** from Bahrain highlighted the importance of educating the editors on ethics. The editors, she opined, must understand morality; they should know their rights, roles and responsibilities. They are also expected to educate the reviewers on what and how they are working. Editors, she concluded, should be honest and when in doubt consult the support staff, organization and the respective institutions.

